# Comparative transcriptome analysis reveals compatible and recalcitrant genotypic response of barley microspore-derived embryogenic callus toward *Agrobacterium* infection

**DOI:** 10.1186/s12870-021-03346-2

**Published:** 2021-12-07

**Authors:** Yingbo Li, Guimei Guo, Hongwei Xu, Ting He, Yingjie Zong, Shuwei Zhang, Muhammad Faheem, Ruiju Lu, Longhua Zhou, Chenghong Liu

**Affiliations:** 1 Biotech Research Institute, Shanghai Academy of Agricultural Sciences/Key Laboratory of Agricultural Genetics and Breeding, Shanghai, China; 2 Nuclear Institute of Agriculture, Tando jam, Pakistan

**Keywords:** Agrobacterium, Transformation, Barley, Microspore-derived embryogenic callus, Genotype, Transcriptome analysis

## Abstract

**Background:**

The *Agrobacterium* mediated transformation has been routinely used in lots of plant species as a powerful tool to deliver genes of interest into a host plant. However, the transformation of elite and commercially valuable cultivar is still limited by the genotype-dependency, and the efficiency of *Agrobacterium* infection efficiency is crucial for the success of transformation.

**Results:**

In this study, the microspore-derived embryogenic calli (MDEC) of barley elite cultivars and breeding lines were employed as unique subjects to characterize the genotypic response during *Agrobacterium* infection process. Our results identified compatible barley genotypes (GanPi 6 and L07, assigned as GP6-L07 group) and one recalcitrant genotype (Hong 99, assigned as H99) for the *Agrobacterium* strain LBA4404 infection using GUS assay. The accumulation trend of reactive oxygen species (ROS) was similar among genotypes across the time course. The results of RNA-seq depicted that the average expressional intensity of whole genomic genes was similar among barley genotypes during *Agrobacterium* infection. However, the numbers of differentially expressed genes (DEGs) exhibited significant expressional variation between GP6-L07 and H99 groups from 6 to 12 h post-inoculation (hpi). Gene ontology (GO) enrichment analysis revealed different regulation patterns for the predicted biological processes between the early (up-regulated DEGs overrepresented at 2 hpi) and late stages (down-regulated DEGs overrepresented from 6 to 24 hpi) of infection. KEGG analysis predicted 12 pathways during *Agrobacterium* infection. Among which one pathway related to pyruvate metabolism was enriched in GP6 and L07 at 6 hpi. Two pathways related to plant hormone signal transduction and DNA replication showed expressional variation between GP6-L07 and H99 at 24 hpi. It was further validated by qRT-PCR assay for seven candidate genes (*Aldehyde dehydrogenase*, *SAUR*, *SAUR50*, *ARG7*, *Replication protein A*, *DNA helicase* and *DNA replication licensing factor*) involved in the three pathways, which are all up-regulated in compatible while down-regulated in recalcitrant genotypes, suggesting the potential compatibility achieved at later stage for the growth of *Agrobacterium* infected cells.

**Conclusions:**

Our findings demonstrated the similarity and difference between compatible and recalcitrant genotypes of barley MDEC upon *Agrobacterium* infection. Seven candidate genes involved in pyruvate metabolism, hormonal signal transduction and DNA replication were identified, which advocates the genotypic dependency during *Agrobacterium* infection process.

**Supplementary Information:**

The online version contains supplementary material available at 10.1186/s12870-021-03346-2.

## Background

Genetic modification through *Agrobacterium* transformation of crops represents the viable solution to address the future food insecurity due to climate change and continual increase in global population. For decades, the *Agrobacterium* mediated transformation remained as a dominant technology for producing genetically modified plants for both basic research and biotechnological applications due to its capacity to transfer low copy number of large segments of DNA into the host genome at a very low cost [1]. Although the *Agrobacterium* mediated transformation has been established successfully in many crops, such as tobacco, soybean, rice, cotton, maize and barley [2], but the high transformation efficiency is limited to a few model genotypes. Genotype is one of the main factors in determining the efficiency of *Agrobacterium* transformation [3]. Due to the different genetic background or cell morphology, different plant species respond differently towards *Agrobacterium* infection. For instance, rice which appears to be the least genotype dependent, even then the transformation efficiency for indica rice is much lower than that for japonica cultivars [4]. Model genotypes are suitable for transformation systems, but in many cases are not desirable for gene evaluation, especially for some elite and commercially valuable cultivars or breeding lines. Therefore, the selection of genotype of the explants is very crucial that can hardly be overcome or complemented through optimizing other external factors.

To date, several studies have been carried out to detect the differential gene expression in response to *Agrobacterium* in plants. Most of the researches aimed to untangle the unique responses related to particular steps of *Agrobacterium* infection by different strains. For example, by comparative analysis of the infection with avirulent or virulent *Agrobacterium* strains in *Arabidopsis*, it was suggested that T-DNA or Vir proteins played crucial role in the plant gene expression [5]. Additionally, few studies discovered the genotypic difference in the responses to *Agrobacterium*. One study showed biological processes related to cell cycle, cell division and DNA repair were overrepresented among the down-regulated genes in recalcitrant genotype at the earliest infection time-points [4]. This study also suggested that degradation of the proteins coating the T-complex might be inhibited in more recalcitrant variety, leading to low transformation frequency. Similarly, another study showed intense defense response of explant is an obstacle for *Agrobacterium* mediated soybean transformation [6]. Most of the researches are in a view that plant host defense might be an important factor influencing the susceptibility of plant cells to *Agrobacterium*. The efficiency of *Agrobacterium* mediated transformation was increased with the addition of plant immune response inhibitor (tenoxicam) in Jatropha and maize [7]. However, tenoxicam didn’t show any effect on the transformation of rice callus. These results advocated the presence of different mechanism between plant tissue and callus. In addition, number of genes important for *Agrobacterium* mediated transformation have been identified in *Arabidopsis* [8]. However, the functions of the most of these genes remained unclear [9]. As the mechanisms of host cells’ response to *Agrobacterium* differ among plant species, therefore there is need to evaluate more plant species to better understand the molecular mechanisms involved in the process [10].

Barley (*Hordeum vulgare* L.), the world’s fourth cereal crops, exhibits strong adaptation to abiotic stresses among cereal crops, and recognized as an ideal model plant to study the mechanism of environmental adaptation for crop improvement. Han et al. [11] reported an efficient method of *Agrobacterium* mediated transformation for five barley cultivars through anther culture. However, the obstacle of genotypic dependency is still required to be overcome for *Agrobacterium* mediated transformation in barley. Microspore embryogenesis emerged as a robust technique to produce double haploids (DHs) in crops [12]. The technique has also provided a model system with uniform, synchronized and easily accessible populations of calli for transformation [12]. In our previous work, we established an efficient protocol of isolated microspore culture for barley [13]. To date, microspore embryogenesis has been successfully carried out in many barley cultivars or breeding lines in our laboratory. The stable *Agrobacterium*-mediated transformation through barley microspore cultures has been reported but the hosts are limited to two barley cultivars ‘Igri’ and ‘Gimpel’ [14, 15]. It is well understood that the efficiency of *Agrobacterium*-mediated transformation in barely is significantly affected by the genotypic response of microspore-derived embryogenic calli (MDEC). Although many studies on the mechanism related to plant genotypic response towards *Agrobacterium* infection have been reported [4], our understanding on the genotype-dependency of transformation is still limited. It is believed that the compressive and extensive researches will pave an avenue to overcome genotypic obstacles in *Agrobacterium* mediated transformation.

In order to establishment of *Agrobacterium* mediated transformation system, we successfully produced large quantity of MDEC from three barley genotypes, GanPi 6 (coded as GP-6),L07 and Hong 99 (coded as H99), using established isolated microspore culture protocols [13]. The explants were further used for *Agrobacterium* mediated transformation. Interestingly, it was found that MDEC from GP-6 and L07 was compatible to infection with *Agrobacterium* strain LBA4404 while MDEC from H99 was recalcitrant to the infection. With the aim to find the reason of genotype-dependency of transformation in barley MDEC. Three barley genotypes were divided into two groups, i.e. compatible group (GP6-L07 group) and recalcitrant group (H99) to characterize the genotypic response during *Agrobacterium* infection process. Comparative transcriptome analysis was performed to profile the expressional patterns between genotypes across time course. Additionally, gene ontology functional analysis and pathway enrichment analysis were deployed to identify the biological processes, pathways and candidate genes involved in the specific genotypic response to the infection process. Our findings disclose the genotypic difference between compatible and recalcitrant explants during infection, and deepen our understanding towards the molecular mechanism of plant response to *Agrobacterium* infection.

## Results

### Variations in susceptibility of three barley genotypes to Agrobacterium infection

**Fig. 1:**
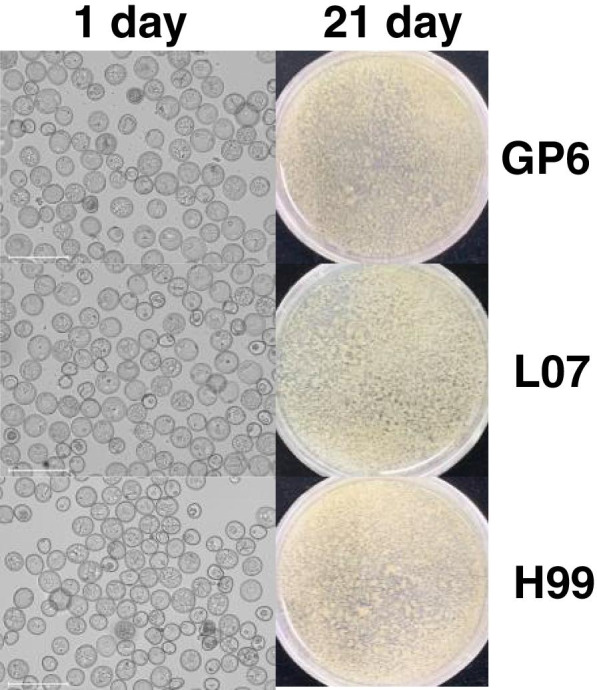
Microspores derived embryogenic callus process of GP6, L07 and H99 at 1 day (single cells) and 21 day (calli). Bars = 125 μm

Three barley genotypes, GanPi6 (GP6), L07 and Hong 99 (H99) from different growing regions of China were used in this study. Microspores isolated from the anthers of these genotypes were cultured under controlled aseptic conditions. After 21 days of culturing, the MDEC were obtained and subsequently used for *Agrobacterium*-mediated transformation (Fig. 1). Infection efficiency was measured to check the compatibility between barley MDEC and *Agrobacterium* strain LBA4404.

**Fig. 2:**
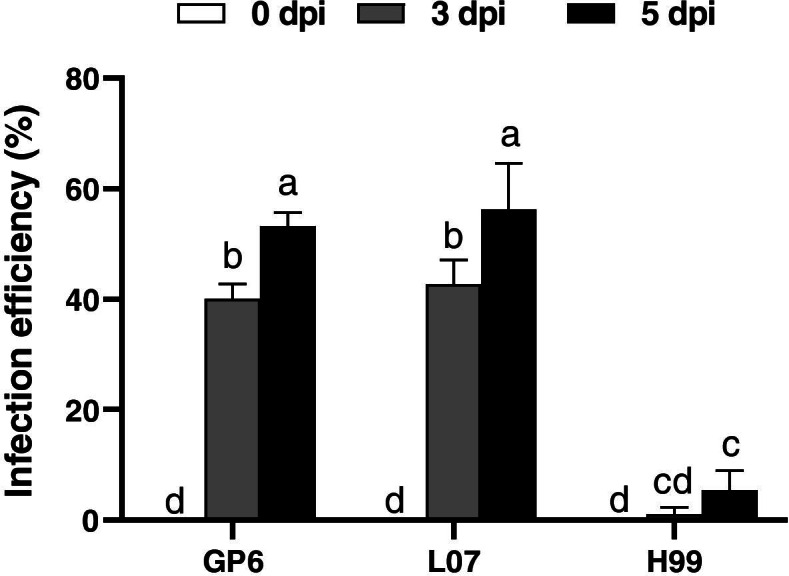
Infection efficiency in three barley genotypes by infection time course. Error bars in the figures indicate standard error. The data are presented as average ± s.e. of three independent samples. Different lowercase letters indicate significant differences (*p* < 0.05) among different genotypes by LSD test

Infection efficiency was measured through transient expression of *GUS* in three barley calli by counting blue spots after 3- and 5- d co-cultivation. As shown in Fig. 2 and supplementary Table S1, infection efficiency of GP6 and L07 was 40.02 and 42.69% at 3 dpi, while it was 0.93% in H99. At 5 dpi, infection efficiency of GP6 and L07 increased to 53.21 and 56.24% respectively while it was calculated as 5.39% in H99.

**Fig. 3:**
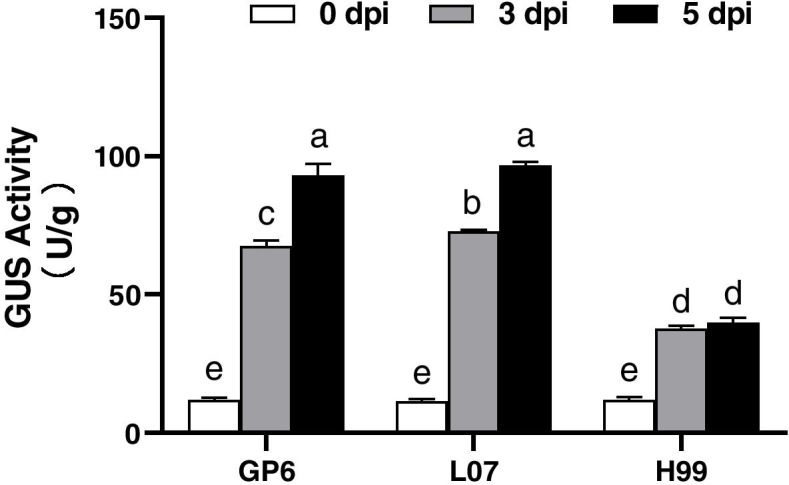
Kinetics of GUS expression in three barley genotypes by infection time course. GUS activity shown represents the average activity ± s.e. of three independent samples. Different lowercase letters indicate significant differences (*p* < 0.05) among different genotypes by LSD test

GUS activity was further measured to verify the GUS staining results. As shown in Fig. 3, GUS activity increased from 3 dpi to 5 dpi in all three barley genotypes, however significantly higher GUS activity was observed in GP6 and L07 than that in H99 at the same time points after infection. These results showed that the cultivars L07 and GP6 were compatible with *Agrobacterium* strain LBA4404, but H99 was recalcitrant.

### Quantitative assay of ROS concentration in three barley genotypes under Agrobacterium infection

**Fig. 4:**
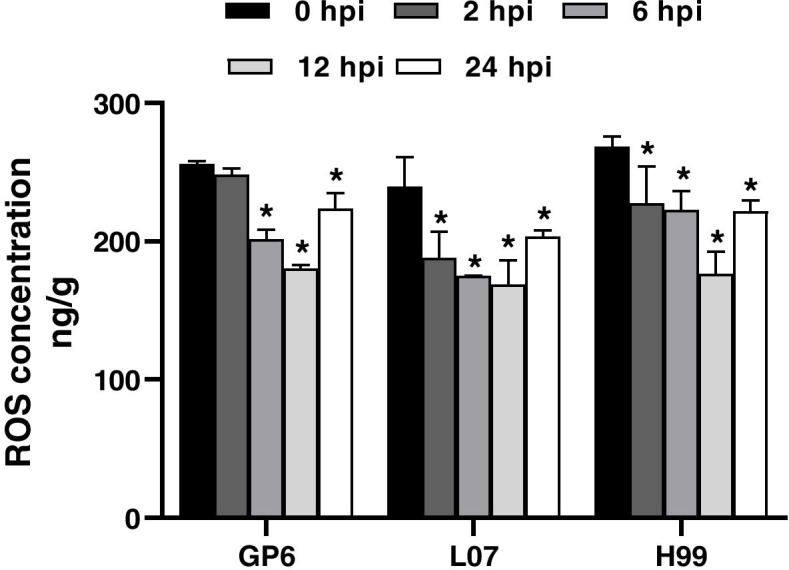
Concentration of ROS in three barley genotypes across infection process. ROS concentration shown represents the average activity ± s.e. of three independent samples

ROS was rapid generation during the bio- or abiotic stress in plant cells, and playing an important role [16]. ROS concentration was measured in three barley genotypes from 0 to 24 h after inoculation with *Agrobacterium*. To our surprise, the scavenging activity of ROS was directly proportional to the passage of infection time in all three barley genotypes (Fig. 4). The highest concentration was found at 0 hpi in each barley genotype. Upon *Agrobacterium* infection, ROS concentration was decreased from 0 to 12 hpi, then increased at 24 hpi. These results indicated barley MDEC suffering stress in the culture environment, and ROS may not the main impact factors related to the infection efficiency of *Agrobacterium* in different genotype of barley MDEC.

### RNA sequencing of three barley genotypes and data analysis

T-DNA was transferred into the host cells of intact plants within 24 h after infection [17]. To investigate the difference in mechanism between compatible and recalcitrant barley genotypes with *Agrobacterium*, RNA was extracted from three barley genotypes inoculated with LBA4404 from 0 to 24 hpi (0, 2, 6, 12 and 24 hpi) for RNA-Seq. Forty-five digital gene expression libraries were constructed using Illumina sequencing platform (Supplementary Table S2). In average, 46.98 million raw reads were generated from each library. After removing the low-quality reads, clean-read numbers per library ranged from 39.58 to 49.64 million. The ‘*Phred* value’ > 30 (Q30) of each library ranged from 91.93 to 94.77%. The number of reads, ranging from 89.52 to 95.64% were mapped to the barley reference genome. The transcriptome data of all samples accomplishing strict quality parameters were further used for bioinformatics analysis.

### Comparative analysis of transcriptomes in three barley genotypes under Agrobacterium infection

**Fig. 5:**
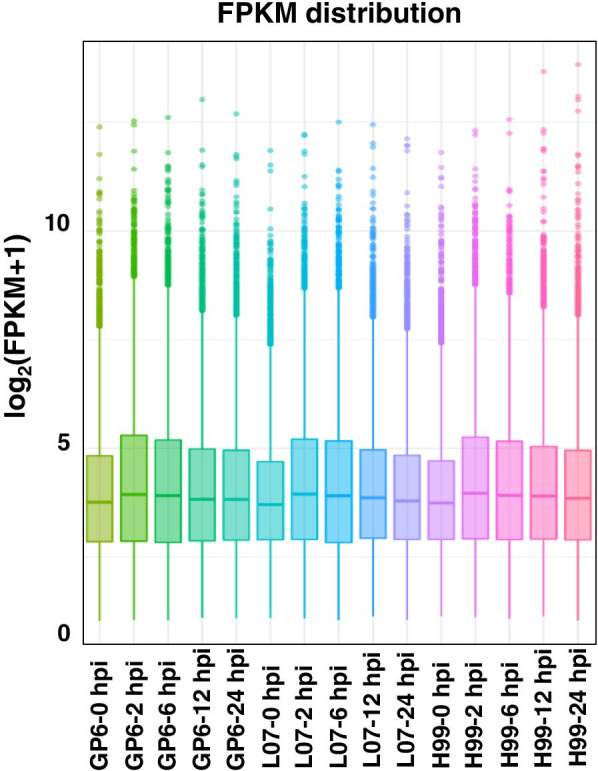
Comparison of whole genomic gene expression for three barley genotypes under *Agrobacterium* infection at five time points (0, 2, 6, 12, and 24 hpi)

Whole genomic gene expression of three barley genotypes during *Agrobacterium* infection at five time points were compared. Comparative analysis results showed that average expressional intensity of whole genomic genes in three genotypes was similar. The average expression level reached highest at 2 hpi, then decreased in the subsequently time points (Fig. 5).

**Fig. 6:**
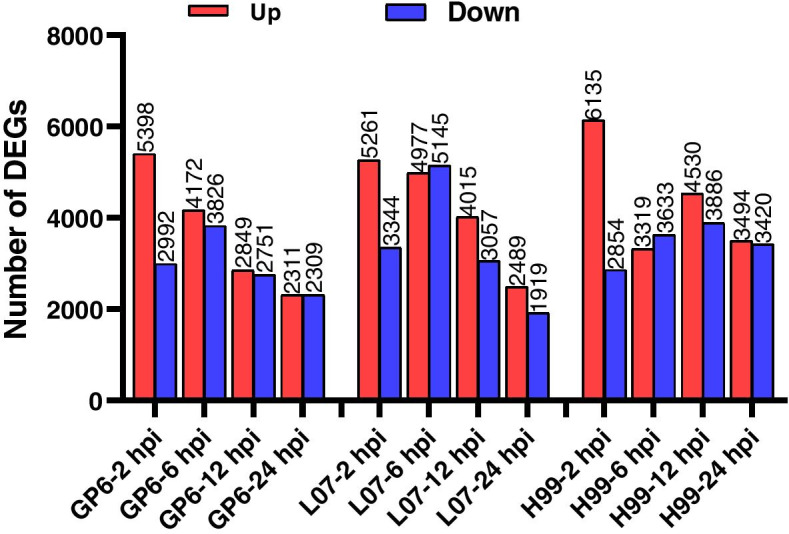
Number of differently expressed genes in three barley genotypes at four time points (2, 6, 12, and 24 hpi) compared with each 0 hpi. The y axis represents DEGs number. Number of DEGs was counted with the criteria *p* < 0.05 and log_2_ (fold change) ≧1. Red column shows up-regulated DEGs, blue column shows down-regulated DEGs

Number of DEGs in three barley genotypes under *Agrobacterium* infection was counted at each time point [0 hpi used as control, *p* < 0.05, and log_2_(fold change≧1)]. Expressional pattern of DEGs was represented as upregulated group and downregulated group at each time point (Fig. 6). A similar trend regarding change in the number of DEGs was observed in GP6 and L07. The number of the upregulated DEGs decreased from 2 hpi to 24 hpi in GP6 and L07, while the number of downregulated DEGs increased from 2 hpi, reached a peak at 6 hpi, and then decreased from 6 hpi to 24 hpi. In H99, the number of upregulated DEGs decreased from 2 hpi to 6 hpi, then increased onward to reach a peak at 12 hpi, and then decreased subsequently from 12 hpi to 24 hpi. Number of the down-regulated DEGs increased from 2 hpi and reached a peak at 12 hpi, then decreased from 12 hpi to 24 hpi. Additionally, the number of DEGs (upregulated DEGs plus downregulated DEGs) was more in H99 than that in GP6 and L07 at each time point except 6 hpi. These results indicated that the response to *Agrobacterium* infection was different between GP6-L07 group and H99.

**Fig. 7:**
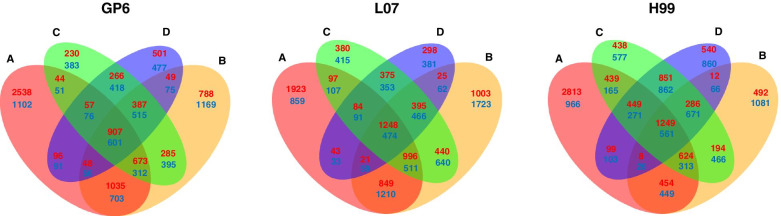
Venn diagram comparison of DEGs at four infection stages (2, 6, 12 and 24 hpi) in three genotype of barley MDEC compared with each 0 hpi. A: DEGs at 2 hpi; B: DEGs at 6 hpi; C: DEGs at 12 hpi; D: DEGs at 24 hpi. Red highlighted numbers represent the amount of upregulated DEGs, and blue highlighted numbers represented the amount of downregulated DEGs

Venn diagrams were further constructed using the number of DEGs in each barley genotype (Fig. 7). In GP6, 2538 upregulated DEGs were specially detected at 2 hpi, which is higher than the numbers (ranging from 230 to 788) detected specially at other time points. At 6 hpi, more downregulated DEGs (1102) were specially detected as compared to at other time points. In total, 907 up- and 601 downregulated DEGs were detected at all four time points. Similar with GP6, 1923 upregulated DEGs were specially detected at 2 hpi in L07. The maximum number of downregulated DEGs were detected at 6 hpi (1723) specially, while 1248 up- and 474 downregulated DEGs were detected at all four time points. In H99, the maximum number of upregulated (2813) and downregulated (1081) DEGs were detected specially at 2 hpi and 6 hpi, respectively. Whereas 1249 up- and 561 downregulated DEGs were detected at all the four time points. These results indicated the diverse response to *Agrobacterium* infection among barley MDEC with different genotype. Additionally, the number of DEGs expressed across each time points varied differently for each genotype of barley MDEC.

**Fig. 8:**
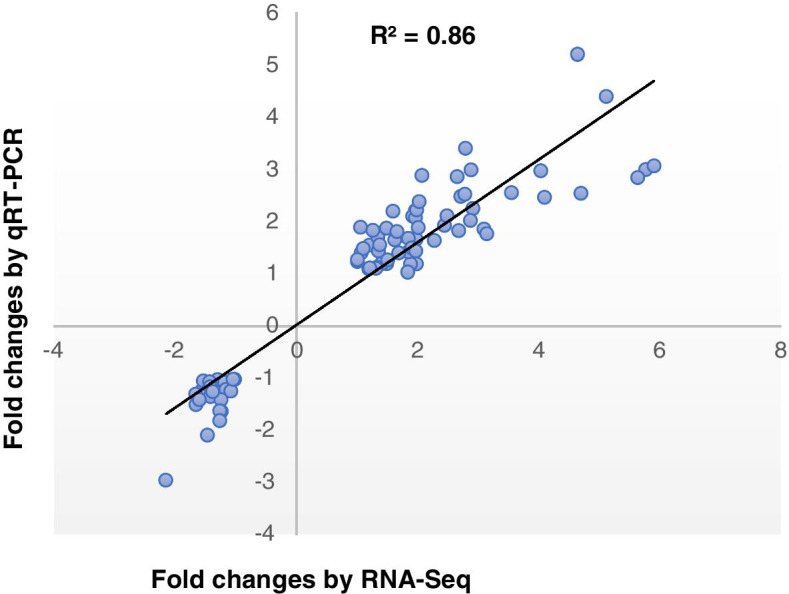
Validation of transcriptome data by qRT-PCR. Scatter plots indicate the transcriptional changes of qRT-PCR analysis and RNA-seq for 81 data points from 12 genes in 12 samples. The genes including *Secretion-associated ras 1*, *Fructose-bisphosphate aldolase 3*, *Isocitrate dehydrogenase*, *Downy mildew resistant 1*, *Embryo defective 2719*, *Phenylalanyl-tRNA synthetase*, *Glutamine synthetase*, *GTP-binding protein SAR1A*, *Glutamyl-tRNA synthetase*, *Proteasome subunit pab1*, *Phosphoribosyl anthranilate isomerase 2* and *Phosphate transporter traffic facilitator1*. The Pearson correlation coefficient (R^2^) was 0.86

To validate the results of the gene expression from RNA-seq data, 12 DEGs were random selected for qRT-PCR analysis (Supplementary Table S3). A strong correlation (*R* ^*2*^ = 0.86) between RNA-seq data and qRT-PCR analysis (Fig. 8) validated the gene expression levels of DEGs.

### Expression pattern of DEGs among three barley genotypes during Agrobacterium infection

**Fig. 9:**
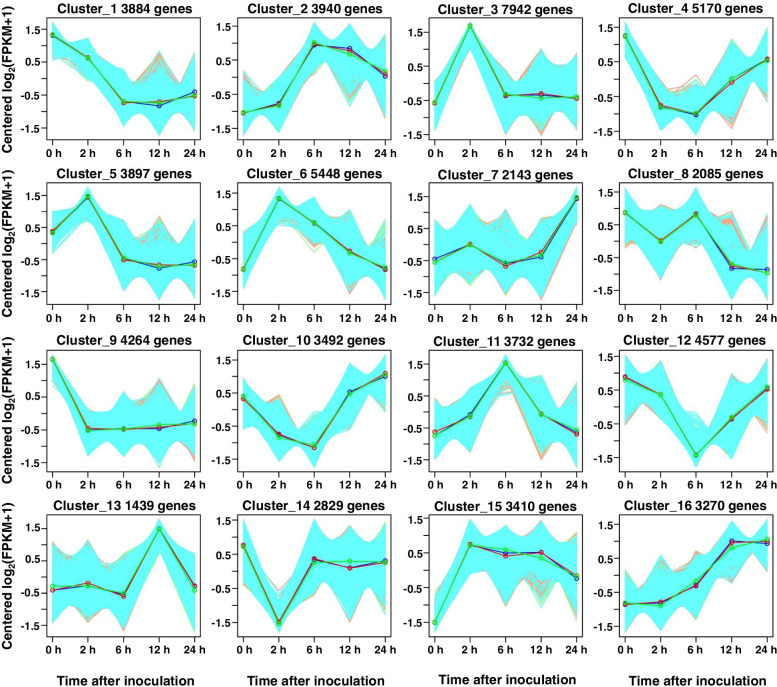
*k*-means clustering by tree-cutting over infection time-course. All DEGs were clustered into 16 groups based on their overall expression profiles. The total number of DEGs falling into each cluster are indicated above each subplot. Green, Blue and red lines represent GP6, L07 and H99, respectively

To group the DEGs based on the expression patterns in compatible and recalcitrant genotype of barley MDEC. We applied the *k*-means clustering on all DEGs at each infection time point. Sixteen clusters were grouped by utilizing the tree-cutting method (Fig. 9). Clusters 1, 4, 8, 9 10, 12, 14 showed a decreased expression pattern in three genotypes response to *Agrobacterium* infection. Clusters 2, 3, 5, 6, 7, 11, 13, 15, 16 showed increased expression pattern. All the clusters showed similar expression patterns in three barley genotypes. This suggested the expression pattern of most DEGs was similar during infection process in three barley genotypes. Genes exhibit different expression pattern at a certain time point may play important roles.

### Comparative GO enrichment analysis of the DEGs in three barley genotypes

**Fig. 10:**
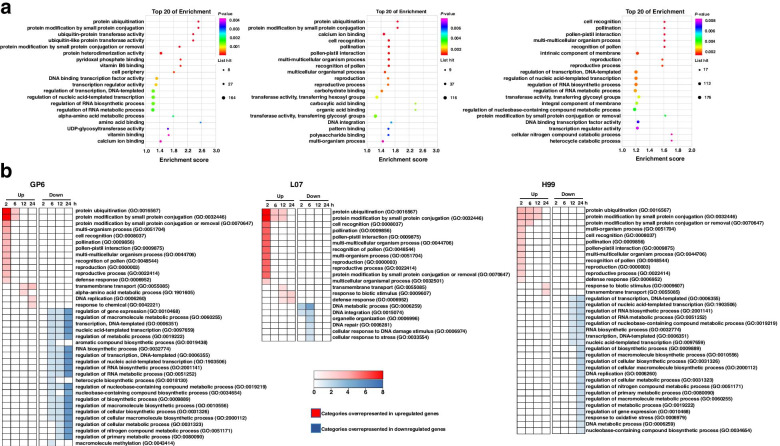
GO analysis of DEGs in three barley genotypes. **a** The selected 20 most enriched GO biological processes categories (*p* < 0.05) among DEGs from different infection timepoints in three barley genotypes. **b** Selected overrepresented GO biological processes categories across time-course comparisons in three barley genotypes. The categories are significantly enriched (*p* < 0.05) among either upregulated or downregulated genes. *p*-values from each category enrichment test have been negatively log10-transformed and plotted for each time point in three barley genotypes

Gene Ontology (GO) functional analysis was deployed by assigning the all DEGs from different time points to evaluate the main biological processes in each barley genotype (*p* < 0.05). The results showed protein ubiquitination was the most common GO category in GP6 and L07, while DEGs in H99 were most involved in cell recognition, multi-multicellular organism and reproductive processes (Fig. 10a, Supplementary data 1). Most of the function categories were same between L07 and H99. To get more information, we further performed GO analysis by assigning the DEGs at each time point (2, 6, 12, 24 hpi). Biological processes were further categorized separately as up-regulated and down-regulated groups (Fig. 10b, Supplementary data 2). The figure showed the selected biological process degree that were overrepresented among the DEGs at each timepoint in three barley genotypes. Most of the biological processes were enriched at 2 hpi among upregulated groups in three barley genotypes. In downregulated groups, most of the function categories were enriched at the infection time points from 6 hpi to 24 hpi in GP6. The function categories were main enriched at 6 hpi in L07. However, most of the function categories were enriched at 24 hpi in H99.

Most of the function categories were same among upregulated groups in three barley genotypes. Categories such as ‘protein ubiquitination’, ‘protein modification by small protein conjugation’ were significantly over-represented among upregulated genes from 2 hpi to 6 hpi in three barley genotypes. ‘multi-organism process’, ‘pollination’, ‘pollen-pistil interaction’, ‘multi-multicellular organism process’, ‘recognition of pollen’ and ‘reproduction’ were significantly over-represented among upregulated genes in three barley genotypes at 2 hpi. ‘Response to biotic stimulus’ and ‘transmembrane transport’ were enriched in H99. The two function categories were also significantly enriched in GP6 or in L07. ‘Defense response’ was significantly over-represented among upregulated genes at 2 hpi in GP6 and H99, whereas it significantly enriched in L07 at 24 hpi.

Among the downregulated biological process groups, most of the function categories were same in GP6 vs H99 at 12 or 24 hpi, such as regulation of gene expression, DNA-templated and nucleic acid-templated transcription, regulation of metabolic (nucleobase-containing compound and nitrogen compound) process, RNA biosynthetic process and metabolic process and regulation of biosynthetic (macromolecule, cellular) process. Interestingly, DNA replication was overrepresented among down-regulated DEGs at 24 hpi in H99, but among up-regulated DEGs in GP6 at 24 hpi. Biological processes that significantly enriched in both GP6 and L07 were same at 6 hpi. The results suggested that most of the biological processes were similar in compatible and recalcitrant genotypes of barley’s MDEC. The same function category with opposite regulated DEGs in compatible and recalcitrant genotype of barley MDEC might be related to *Agrobacterium* infection efficiency.

### KEGG analysis of the DEGs in three barley genotypes

To further understand the different biological pathways in compatible and recalcitrant genotype of barley MDEC during *Agrobacterium* infection. Kyoto Encyclopedia for Genes and Genomes (KEGG) enrichment analysis was performed using the DEGs at each time point (2, 6, 12, 24 hpi) in three barley genotypes. Firstly, H99 was compared with GP6 and then with L07. Same pathways were selected and combined form the two comparisons. This strategy might have missed some pathways, but this was counterbalanced by the reliability of digging pathways specific to the infection efficiency.

Based on this strategy, a comprehensive analysis was performed among three barley genotypes to predict 12pathways (Table 1, Supplementary data 3). Most of the annotated pathways were same among three barley genotypes during *Agrobacterium* infection. ‘plant-pathogen interaction’, ‘MAPK signaling pathway-plant’, ‘phenylpropanoid biosynthesis’ and ‘phenylalanine, tyrosine and tryptophan’ biosynthesis were found upregulated at early stages of *Agrobacteriu*m infection (2 hpi) in all the barely genotypes. Two upregulated pathways ‘carbon metabolism’ and ‘citrate cycle (TCA cycle)’ were upregulated as early as 6 hpi in all the barley genotypes. ‘Pyruvate metabolism’ was upregulated at 6 hpi in GP6 and L07. Three pathways ‘pyruvate metabolism’, ‘glycine, serine and threonine metabolism’, ‘glutathione metabolism’ were all upregulated at 12 hpi. ‘plant hormone signal transduction’ were down-regulated as early as 2 hpi in all three barley genotypes. However, genes in this pathway were upregulated in GP6 and L07, downregulated in H99 at 24 hpi. DNA replication was upregulated in GP6 but downregulated in H99 at 24 hpi.

**Table 1:** DEGs enriched on the pathways in three barley MDEC at 2, 6, 12 and 24 hpi. ≥ 1 means the number of upregulated gene involves in the pathways at each time point, ≤ − 1 means the number of downregulated gene involves in the pathways at each time point

Pathways	2 hpi						6 hpi						12 hpi						24 hpi					
	GP6		L07		H99		GP6		L07		H99		GP6		L07		H99		GP6		L07		H99	
	≥1	≤ − 1	≥1	≤ − 1	≥1	≤ − 1	≥1	≤ − 1	≥1	≤ − 1	≥1	≤ − 1	≥1	≤ − 1	≥1	≤ − 1	≥1	≤ − 1	≥1	≤ − 1	≥1	≤ − 1	≥1	≤ − 1
Plant-pathogen interaction	43		44		44				35		27				24		28			15				
MAPK signaling pathway - plant	24		26		26				23								19							
Phenylpropanoid biosynthesis	25		24		29		23		29		19		20		29		29		16		19		17	
Phenylalanine, tyrosine and tryptophan biosynthesis	12		12		15		15		19		15		9		15		14							
Biosynthesis of amino acids							44		51		38		42		49		41		26		24			
Carbon metabolism							44		43		37		41		47		46		34		25		35	
Citrate cycle (TCA cycle)							17		17		15		15		13		13		15					
Pyruvate metabolism							19		18				16		14		16		16					
Glycine, serine and threonine metabolism							13						10		12		13							
Glutathione metabolism							17						12		17		18						14	
Plant hormone signal transduction		19		21		18							22	16		20		25	20	15	19			28
DNA replication																			14					12

### Identification of candidate genes related to barley MDEC susceptibility to Agrobacterium

**Fig. 11:**
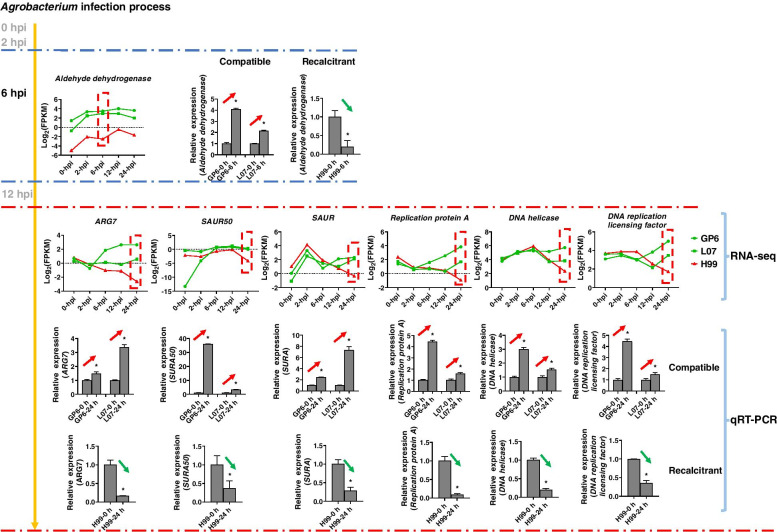
A putative reason for the different infection efficiency by *Agrobacterium* between compatible and recalcitrant barley MDEC. Line graphs at the top indicate log_2_ of FPKM for four identified genes in three barley genotypes across infection process. Histograms represent the validation of seven selected genes in three barley MDEC by quantitative real-time PCR. The expression levels on the y-axis were relative to the non-inoculated sample of after normalization with the barley *Actin* gene. The data were presented as average ± S.D. with *n* = 3. ‘*’ indicates significant differences at the level of *p* < 0.05. Red arrows indicate upregulation, green arrows indicate downregulation

KEGG enrichment analysis showed ‘pyruvate metabolism’ was upregulated pathways in GP6 and L07 at 6 hpi. While ‘plant hormone signal transduction’ and ‘DNA replication’ are oppositely regulated in GP6 and H99 at 24 hpi. This indicated these pathways may play important roles in the *Agrobacterium* infection process. Genes involved in the pathways were further identified in three barley genotypes. Genes exhibited opposite regulation between GP6-L07 and H99 were analyzed. A putative candidate gene was identified from pyruvate metabolism pathway. The gene encoded an aldehyde dehydrogenase (*HORVU0Hr1G031700*), which exhibited more transcript accumulation in GP6 and L07 compared to H99 at 6 hpi (Fig. 11). Three putative candidate genes were identified from plant hormone signal transduction pathway, which showed upregulation in GP6 and L07, but downregulation in H99 at 24 hpi. These genes included two small auxin-up RNA genes (*SAUR*, *HORVU7Hr1G096870* and *SAUR50*, *HORVU7Hr1G017790*), one auxin induced protein (*ARG7*, *HORVU2Hr1G110460*). All the genes belonged to auxin response gene family. Three genes were identified from DNA replication pathway, which showed upregulation in GP6 and downregulation in H99, but exhibited high transcript level in L07 only at 24 hpi. These genes included *Replication protein A* (*HORVU4Hr1G067940*), *DNA helicase* (*HORVU1Hr1G063700*) and *DNA replication licensing factor* (*HORVU1Hr1G070110*). The expressional profile of these genes is represented in Fig. 11 which represented that all these genes exhibited more transcript accumulation in GP6 and L07compared to H99 at 24 hpi (Fig. 11). qRT-PCR assay confirming that all these seven genes were upregulated in compatible genotypes while downregulated in recalcitrant genotype (Fig. 11).

### Expression of Gus gene in three barley genotypes at 24 hpi

**Fig. 12:**
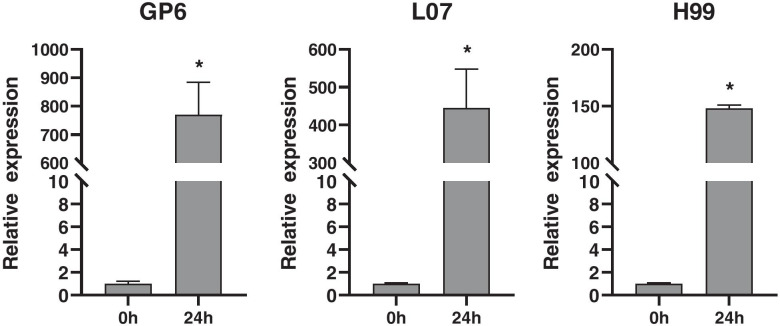
Expression of *Gus* in three MDEC at 0 and 24 hpi*.* The expression levels on the y-axis were relative to 0 hpi after normalization with the barley *Actin* gene. The data were presented as average ± S.D. with n = 3. ‘*’ indicates significant differences at the level of *p* < 0.05

The expression of foreign *Gus* was further determined by qRT-PCR in three barley genotypes at 24 hpi. As shown in Fig. 12, when compared with the expression level at 0 hpi, the relative expression level of *Gus* in GP6 and L07 was more 400 times, while less than 150 times in H99. This suggested more transcript of *Gus* was accumulated in GP6 and L07 as compared to H99.

## Discussion

### Genotype affected the infection efficiency in barley MDEC

*Agrobacterium* mediated transformation has been widely used in plant for decades, however, the genotypic dependency remained the main limiting factor in *Agrobacterium* infection. In the present study, GP6 and L07 MDEC showed high infection efficiency upon *Agrobacterium* infection, but H99 was recalcitrant to *Agrobacterium* infection, because of the low infection efficiency. These results are in consistent to the view that genotype affects the infection efficiency of *Agrobacterium*.

### Global gene expression showed similarly changing trend among barley genotypes upon *Agrobacterium* infection

RNA-seq data from just two materials with contrasting response to *Agrobacterium* may not represent the actually different mechanism. We used three barley varieties (two compatible and one recalcitrant) in the present experiment. The results showed that the *Agrobacterium* infection brought rapid changes in gene expression at early stage of infection (2 hpi) in all the three studied barley genotypes (Fig. 5), moreover, the expressional intensity of all genes was similar in three barley genotypes. This indicated that the MDEC with different genotypes have response upon *Agrobacterium* infection. Jiang et al. [18] showed the early activation (3–6 h) of defense related genes in tobacco BY2 cell suspensions when co-cultivated with *Agrobacterium*. In *Arabidopsis*, the host defense response was stimulated by agrobacterial strains as early as 3 hpi [19]. Similarly, in rice embryogenic calli gene expression changes were detected as early as 1 hpi upon *Agrobacterium* infection [4]. These studies suggested that the response of different plant species to *Agrobacterium* is discrepancy and subsequently influence the transcripts in different plants. In rice, more DEGs in *Indica* rice variety Zhen Shan 97 were observed as compare to that in *Japonica* cultivars Nipponbare upon *Agrobacterium* infection [4]. In our study, more DEGs were detected in H99 as compared to GP6 and L07 during *Agrobacterium* transformation except at 6 hpi (Fig. 6). The results are in consistent with rice, because both H99 and Zhen Shan 97 were found to be *Agrobacterium* transformation recalcitrance.

The results of transcriptome analysis showed similar expression patterns in all the three barley genotypes upon *Agrobacterium* infection. Tie et al. [4] showed that most of the gene expression profiles were similar in Nip and Zhen Shan 97 calli after *Agrobacterium* infection. Duan et al. [5] showed that the expression of DEGs were similar in *Arabidopsis* seeding whether the *Agrobacterium* strains transfer T-DNA in the plant cell. Our results were in line with the previous findings. *A. tumefaciens* is plant pathogen that causes crown gall disease [20], meanwhile, the plant has evolved sophisticated defense system for the *Agrobacterium* infection. All these studies provide strong evidence that same transcripts were involved during *Agrobacterium* infection but the intensity of these transcript varied according to plant species as well as on genotypes within the same species.. The variation in gene expression pattern at a certain time point may affect the infection of *Agrobacterium*.

GO enrichment analysis was first performed on each barley genotype by using all DEGs from different time points. The common biological processes between GP6 and L07 were protein modification and protein ubiquitination. However, most of the biological processes were similar between L07 and H99. GO enrichment analysis was further performed on three barley genotypes at each infection time point. Most of the significant enriched biological processes were same in all the barley genotypes. Which suggest the involvement of few biological processes in the infection efficiency. Certain biological process like protein modification process such as ‘small protein conjugation or removal’ and ‘protein ubiquitination’ were biological processes among upregulated DEGs in three barley genotypes upon *Agrobacterium* infection. Protein ubiquitination is involved in various developmental processes in plants, such as hormone regulation, photomorphogenesis, floral homeostasis, embryo development and defense response [21]. The process was also necessary for the *Agrobacterium* mediated transformation [22]. In wheat callus, ubiquitin mediated proteolysis pathway was also upregulated under *Agrobacterium* infection [23]. However, Tie et al. [4] showed some genes involved in ubiquitin proteasome system were down-regulated in recalcitrant callus. This is different from our findings, because there was no difference between compatible and recalcitrant genotype of barley MDEC.

### Defense response couldn’t affect the infection efficiency in barley MDEC

KEGG enrichment analysis of barley revealed the involvement and up-regulation of genes related to plant defense during *Agrobacterium* infection in all three barley genotypes. Plant defense response to pathogen was the common process or pathway that were found in rice, wheat, and Arabidopsis under *Agrobacterium* infection [4, 5, 23]. This reflected the constant battle between *Agrobacterium* and the host plant cell. Tie et al. [4] showed the repression of some defense related genes in the transformation of compatible callus. This is different from our results, which showed no difference in three barley genotypes. The quantitative assay of ROS concentration further proved our results. Oxidative burst is the first defense of plants against pathogen attacks [24]. The changing trend of ROS concentration was similar in three barley genotypes upon *Agrobacterium* infection. This reflected that the defense response was similar in all three barley genotypes under *Agrobacterium* infection. Moreover, the addition of plant immune response inhibitor (tenoxicam) couldn’t increase the transformation efficiency in rice callus [7]. Taken together, these results presented that defense response to *Agrobacterium* infection was commonly in different genotype of barley MDEC, which may did not affect the infection efficiency.

### Exploration of pathways and genes that affected the infection efficiency in barley MDEC

KEGG enrichment analysis showed pyruvate metabolism was upregulated pathway that enriched in compatible genotypes at 6 hpi. Pyruvate metabolism was one of the basic metabolisms in plant cells. Gonzalez-Mula et al. [25] showed that *A. tumefaciens* could use plant metabolites as nutrients. Pyruvate metabolism acted as a node connecting the pathways such as tricarboxylic acid (TCA) cycle and gluconeogenesis [25]. One candidate gene (*Aldehyde dehydrogenase*) involved in pyruvate metabolism was identified. The gene was up-regulated in the compatible while down-regulated in the recalcitrant genotype at 6 hpi. Aldehyde dehydrogenase was showed that supported glycolysis and TCA cycle in mammalian cells [26]. The upregulated of *Aldehyde dehydrogenase* may promoted the production of metabolites in MDEC, and enhanced the growth of *Agrobacterium*.

Genes involved in plant hormone signal transduction and DNA replication were upregulated in compatible genotypes, but down regulated in recalcitrant genotype at 24 hpi. Agrobacteria shift the hormone balance in their infected host cells to promote the tumor formation [27]. Thus, plant hormone related genes always be induced upon the *Agrobacterium* infection [4, 19]. In rice, genes involved in response to hormone stimulus were upregulated in Nip callus at 24 hpi. Similarly, we found that three auxin related genes (*SAUR*, *SAUR50* and *ARG7*) were up-regulated in the compatible while down-regulated in the recalcitrant genotype. Auxin involved in all aspects of plant growth as well as cell division [28], which was overproduced in *Agrobacterium* infected cells, and contributed to tumor growth [29]. Veena et al. [30] showed that genes associated with cell division and growth processes were induced during later times of infection (18–36 h) in tobacco cells. Two studied showed that numerous auxin related genes were induced during callus formation, either in barley microspores or in embryos [31, 32]. The upregulation of auxin related genes suggested the growth of calli cells. In addition, *SAUR* function as positive effector of cell expansion through the modulation of auxin transport [33]. The growth of *Agrobacterium* infected cells may result in more cells with foreign *Gus* gene, and finally help increasing the infection efficiency.

Tie et al. [4] showed that genes involved in cell cycle and division were repressed early after transformation in recalcitrant callus but up-regulated during the later stages of transformation in compatible callus. This is similar with our results. Interestingly, genes involved in DNA replication process were downregulated at early time (0–2 d) during barley microspore culture, while upregulated at later stage (2–5 d) [31]. This suggested DNA replication might be a regulator contributing to the callus formation. DNA replication process functions in S phase of the cell cycle, which is essential for *Agrobacterium* mediated transformation [34, 35]. Three candidate genes involved in DNA replication were identified. Higher transcript level of the genes was observed in compatible genotypes than that in recalcitrant genotype at 24 hpi. All the genes (*Replication protein A*, *DNA helicase* and *DNA replication licensing factor*) have been proved control the cell proliferation and growth in plant [36–38]. Replication protein A is required for multiple processes in DNA metabolism such as replication, repair, and homologous recombination. Dafny-Yelin et al. [39] showed overexpression of three yeast DNA replication factor A protein in tobacco blocks single-stranded DNA conversion into double-stranded. However, *DNA replication A* was upregulated at 24 hpi in the compatible genotypes while downregulated in recalcitrant genotype (Fig. 11). Narasimhulu et al. [17] found T-DNA was transferred into the host cells of plants as early as 18 h. The upregulated of *replication protein A* in compatible barley MDEC may occurred after *Agrobacterium* mediated transformation. As a necessary factor to form and maintain artificial chromosomes, DNA helicase gene SRS2 was important in efficient *Agrobacterium*-mediated yeast transformation with chromosomal T-DNA [40]. In *Arabidopsis*, the overexpression of DNA replication licensing factor (CDT1a) can stimulate DNA replication [41]. DNA replication is also indispensable for the DNA repair machinery, in which process T-DNA molecules is replicated in the host cells [42]. We further determined the expression of foreign *Gus* gene in three barley genotypes at 24 hpi. The transcript accumulation of *Gus* in GP6 and L07 was more than that in H99 (Fig. 12). Taken together, we speculated the upregulated of pyruvate metabolism, auxin related and DNA replication pathways may enhance growth of *Agrobacterium* and infected cells during infection process, finally resulted in high transcript accumulation of *Gus*.

## Conclusions

Taken together, this study clearly demonstrated the similarity and difference between compatible and recalcitrant genotype of barley MDEC upon *Agrobacterium* infection. During the *Agrobacterium* infection, the accumulation trend of ROS, and the expressional intensity and patterns of whole genomics were similar among three genotypes. We found that seven candidate genes (*Aldehyde dehydrogenase*, *ARG7*, *SAUR*, *SAUR50*, *Replication protein A*, *DNA helicase* and *DNA replication licensing factor*), which is related to plant hormone signal transduction and DNA replication, were up-regulated only in compatible genotypes. The higher transcript accumulation of *Gus* reveals that the potential compatibility achieved at later stage for the growth of *Agrobacterium* infected cells. The findings will provide additional insights of the molecular events occurring during the process of *Agrobacterium*-mediated transformation, and will help to expand the potential for improving recalcitrant genotype of barley MDEC transformation.

## Materials and methods

### Plant materials and microspore culture

Barley (*Hordeum vulgare* L.) genotypes were selected from three cultivation zones of China. Barley *cv*. GanPi6 is an elite malting cultivar from Gansu province, the northwest of China. The cultivar was created by Gansu Academy of Agricultural Sciences. Hong99 is an excellent barley breeding line from Heilongjiang province, the northeast of China. The material was created by Hongxinglong institute of agricultural sciences. L07 is an excellent breeding line from Shanghai, the east of China. The material was created by Shanghai Academy of Agricultural Sciences. All the materials were premised to use in this research. And the seed of three barley materials were preserved in Shanghai Academy of Agricultural Sciences. The regeneration rate (plants/100 g callus) of three genotypes is promising and all show high responsive for green plant regeneration: GanPi6 (216), Hong99 (160.7) and L07 (191.3). All the materials were grown at a controlled environment room. The growth conditions are: 20/16 °C (day/night) with a 12 h photoperiod, 20,000 lx, 50% humidity. Microspore culture was raised as previously described by Lu et al. [13]. Briefly, the collected spikes were subjected to cold pretreatment at 4 °C for 2 weeks, and the microspores were collected by crushing the anthers from the sterilized spikes. The collected microspores were isolated by filtration and centrifugation, and finally cultured on the induction medium (N6 basal medium supplemented with 2.0 μM 2.4-D, 2.3 μM Kinetin, and 0.25 M maltose) for embryogenic callus induction. After 21 d of culturing, the microspore-derived embryogenic calli (MDEC) were used for *Agrobacterium* mediated transformation.

### Agrobacterium infection on barley MDEC

*Agrobacterium* strain LBA4404, harboring binary vector *pCAMBIA1305.1,* was used for barley transformation of MDEC. Overnight cultures of *Agrobacterium* (~ 0.6 at OD600) in YEP medium were pretreated with 200 μM acetosyringone, in an incubator shaker at 28 °C with shaking at 250 rpm for 2 h, 28 °C. The bacteria were then collected by centrifugation at 3270 g for 15 min, and resuspended in *Agrobacterium* suspension medium (Induction medium with 200 μM acetosyringone) to a final optical density at 600 nm (~ 0.8 at OD600). The bacterial suspension was kept in an incubator shaker 28 °C with shaking at 80 rpm, for about 30 min prior to use.

For Agroinfection experiment, the barley MDEC (more than 100 calli) were infected with 20 ml of *Agrobacterium* suspensions with slight agitation (100 rpm) for 30 min and then blotted dry with three layers of sterile filter paper for 30 min to remove excess *Agrobacterium*. Co-cultivation was performed in a petri dish (10 cm) by placing the infection calli on two layers of filter paper, which is pre-soaked with 5 ml liquid induction medium containing 200 μM acetosyringone. The petri dishes sealed with Parafilm, then put in a growth chamber for culture at 23 °C. Samples were harvested at five different time points (0, 2, 6, 12, 24 h post-inoculation, hpi). Three replicates were performed for each time point.

### Assay of GUS activity and ROS production

GUS stain was performed at 3 and 5 days after co-cultivation with *Agrobacterium*. Callus pieces were randomly picked to stain with 0.2 mg/L 5-bromo-4-chloro-3-indolyl glucuronide (X-Gluc) solution for GUS activity at 37 °C overnight. Then the blue staining was examined visually and using stereo microscope to calculate the efficiency of transient transformation in three biological replicates. For one biological replicate, a petri dish of calli (more than 100) was stained and calculated. The frequency of transformation was calculated as follows: [(Number of GUS^+^ calli)/(Total number of calli inoculated with *Agrobacterium*)] × 100%.

The kinetics of GUS and reactive oxygen species (ROS) production in the calli were measured by GUS and ROS Elisa kit (Kejing Biological Technology, Jiangsu, China) according to the manufacturer’s protocols. For GUS assay, different genotype of barley MDEC were collected after 3- and 5- d co-cultivation with *Agrobacterium*. For ROS assay, the MDEC were collected at 0, 2, 6, 12 and 24 h after co-cultivation with *Agrobacterium*. 0.1 g MDEC was weighed from each petri dish and used for GUS and ROS experiment, respectively. Three biological replicates were performed with each barley MDEC. Standard deviations and a paired sample *t*-test for statistical analysis were performed using the SAS software.

### RNA extraction and preparation of cDNA library

The total RNA of *Agrobacterium* infection MDEC samples was extracted using TRIzol regent (Invitrogen, Carlsbad, CA, USA) according to the manufacturer’s protocol. RNA integrity was confirmed using the 2100 Bioanalyzer (Agilent Technologies, Palo Alto, CA, USA). RNA libraries for transcriptome sequencing were constructed according to the IIIumina RNA Seq library kit (Illumina, Inc.). The total RNA was digested by *DNase* I. mRNA containing poly-A tail was enriched by Oligo (dT) attached to magnetic beads, following by random fragmentation of mRNA into small segments. The first and the second strand cDNA were synthesized using the fragments as templates then followed by end repairing. The ends of DNA fragments were modified and ligated with adapters, and the cleaned ligation products (300–350 bp) were enriched by the PCR (15 cycles) with random primers (random hexamers), following by gel purification. Amplified libraries were checked by the Agilent 2100 Bioanalyzer (Agilent, Inc.).

### RNA sequencing and data analysis

RNA sequencing was performed using Illumina HiSeq™ 4000 platform (Illumina, Inc.) for 150 bp paired-ends sequencing in Bei Jing Novogene Biotech Co., Ltd. Quality controlled (QC) for the raw data were trimmed by removing all empty and low-quality reads (Q < 30 and length < 50 bp), as well as all adaptor sequences in order to obtain clean reads. Putative transcripts annotations were identified by searching the listed annotations of high confidence (HC) genes (2016) (http://webblast.ipk-gatersleben.de/barley_ibsc/downloads/Hv_IBSC_PGSB_r1_HighConf.gtf.gz). The levels of gene expression were calculated by fragments Per Kilo bases per Million reads (FPKM) using the reads mapped to the reference sequence. The resulting *p*-values were adjusted using the Benjamini and Hochberg’s approach for controlling the false discovery rate. Genes with an adjusted *P* value < 0.05 was set as the threshold for significantly differential expression.

GO analysis was based on the website (http://geneontology.org/). Metabolic and cellular pathways were predicted by Kyoto Encyclopedia for Genes and Genomes (KEGG) mapping [43]. Venn diagrams were generated using the online tool NovoMagic (https://magic.novogene.com/) by pasting sets of DEGs in each comparison into the webpage. *k*-means cluster were generated by converting read count values for each DEGs to FPKM in EdgeR and feeding them into the clustering function of the package Trinity RNA seq [44]. Clusters were determined by the tree-cutting method.

### Quantitative RT-PCR analysis

To validate the expressions of DEGs, 12 genes were used for a qRT-PCR analysis. The sequences of these candidate DEGs were obtained from the website (http://plants.ensembl.org/Hordeum_vulgare/Info/Index), and the primer pairs were designed using Primer3 (http://www.premierbiosoft.com/) according to the reference sequences. The selected gene name and primer information is listed in Supplementary Table S4. First-strand cDNA was synthesized from about 1 μg of total RNA using Super Script™ reverse transcriptase (Takara, Dalian, China). The amplification reactions were performed in the ABI 7500 fast instrument (Applied Biosystems, USA) and the SYBR Premix Ex Taq™ Kit (Life, USA) were used following the manufacturers’ instructions. The house-keeping gene of *beta-actin* in barley was used as internal control [45]. The comparative CT method (^△△^CT method) of quantification was used to quantify the relative expression of specific genes [46].

## Supplementary Information

**Additional file 1: Supplementary Table 1.** Infection efficiency in three barley genotypes. **Supplementary Table 2.** An overview of sequencing and assembly of three barley MDEC. **Supplementary Table 3.** Validation of the transcriptome data by qRT-PCR. **Supplementary Table 4.** Primer information for qRT-PCR. **Supplementary Data 1.** Data of significant enriched Biological Process of GO categories in three barley MDEC. **Supplementary Data 2.** Data of significant enriched Biological Process of GO categories across time-course comparisons in three barley MDEC. The categories are separated as upregulate and downregulate groups. **Supplementary Data 3.** Data of pathways by KEGG enrichment in three barley MDEC at 2, 6, 12 and 24 hpi. The pathways are separated as upregulate and downregulate groups.

## Data Availability

The datasets generated and analyzed during the current study are available in the National Center for Biotechnology Information. The raw data for RNA-seq can be downloaded at a https://www.ncbi.nlm.nih.gov/sra/PRJNA749617.

## Abbreviations

MDEC: Microspore-derived embryogenic calli
DH: Doubled haploid
HPI: Hours post inoculation
ROS: Reactive oxygen species
FPKM: Fragments per kilo bases per million
DEGs: Differentially expression genes
GO: Gene ontology
KEGG: Kyoto Encyclopedia for Genes and Genomes
SAUR: Small auxin-up RNA gene
TCA: Tricarboxylic acid
